# Three-stage segmentation of lung region from CT images using deep neural networks

**DOI:** 10.1186/s12880-021-00640-1

**Published:** 2021-07-15

**Authors:** Michael Osadebey, Hilde K. Andersen, Dag Waaler, Kristian Fossaa, Anne C. T. Martinsen, Marius Pedersen

**Affiliations:** 1 Department of Computer Science, Norwegian University of Science and Technology, Gjøvik, Norway; 2 Department of Diagnostic Physics, Oslo University Hospital, Oslo, Norway; 3 Department of Health Sciences, Norwegian University of Science and Technology, Gjøvik, Norway; 4 The Faculty of health sciences, Oslo Metropolitan University, Oslo, Norway; 5 Sunnaas Rehabilitation Hospital, Nesoddtangen, Norway

**Keywords:** Computed tomography (CT), Three-stage segmentation, Deep learning network, Lung region, Lung contour

## Abstract

**Background:**

Lung region segmentation is an important stage of automated image-based approaches for the diagnosis of respiratory diseases. Manual methods executed by experts are considered the gold standard, but it is time consuming and the accuracy is dependent on radiologists’ experience. Automated methods are relatively fast and reproducible with potential to facilitate physician interpretation of images. However, these benefits are possible only after overcoming several challenges. The traditional methods that are formulated as a three-stage segmentation demonstrate promising results on normal CT data but perform poorly in the presence of pathological features and variations in image quality attributes. The implementation of deep learning methods that can demonstrate superior performance over traditional methods is dependent on the quantity, quality, cost and the time it takes to generate training data. Thus, efficient and clinically relevant automated segmentation method is desired for the diagnosis of respiratory diseases.

**Methods:**

We implement each of the three stages of traditional methods using deep learning methods trained on five different configurations of training data with ground truths obtained from the 3D Image Reconstruction for Comparison of Algorithm Database (3DIRCAD) and the Interstitial Lung Diseases (ILD) database. The data was augmented with the Lung Image Database Consortium (LIDC-IDRI) image collection and a realistic phantom. A convolutional neural network (CNN) at the preprocessing stage classifies the input into lung and none lung regions. The processing stage was implemented using a CNN-based U-net while the postprocessing stage utilize another U-net and CNN for contour refinement and filtering out false positives, respectively.

**Results:**

The performance of the proposed method was evaluated on 1230 and 1100 CT slices from the 3DIRCAD and ILD databases. We investigate the performance of the proposed method on five configurations of training data and three configurations of the segmentation system; three-stage segmentation and three-stage segmentation without a CNN classifier and contrast enhancement, respectively. The Dice-score recorded by the proposed method range from 0.76 to 0.95.

**Conclusion:**

The clinical relevance and segmentation accuracy of deep learning models can improve though deep learning-based three-stage segmentation, image quality evaluation and enhancement as well as augmenting the training data with large volume of cheap and quality training data. We propose a new and novel deep learning-based method of contour refinement.

## Introduction

**Fig. 1:**
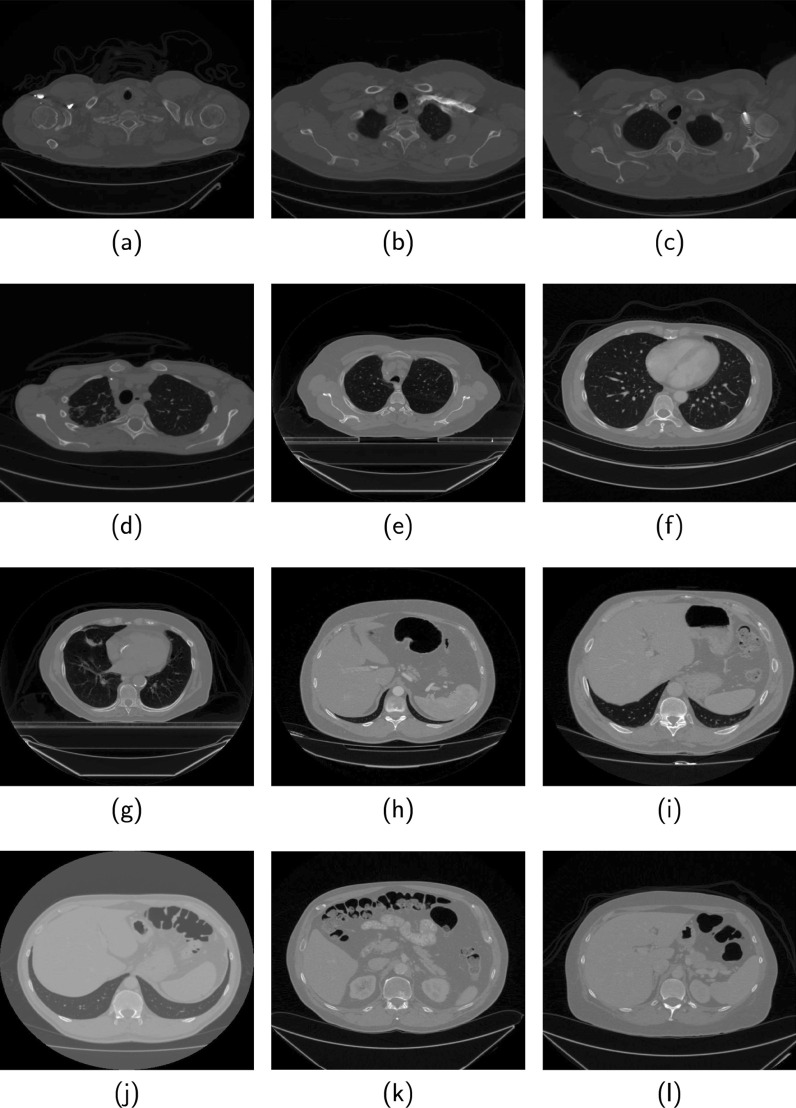
Examples of CT slices from [24, 25] that are acquired during CT lung screening. The slices are arranged successively such that (**a**) is the most superior and (**l**) is the most inferior within the thorax region. Slices in (**a**), (**k**) and (**l**) do not contain lung region

Lung region segmentation is in the early stage of image-based approaches for early detection, diagnosis and treatment of respiratory diseases [1]. Lung cancer, chronic bronchitis and the recent coronavirus disease (COVID-19) are examples of respiratory diseases. The accuracy of subsequent stages of the imaging workflow leading to a reliable diagnosis of respiratory diseases is strongly dependent on accurate segmentation of the lung region. Computed tomography (CT) imaging is widely used for the diagnosis of respiratory diseases because of its relatively small acquisition time and potential to generate high contrast images of the thoracic cavity. Figure 1 are examples of some of the axial slices acquired during a typical CT examination. The slices (Fig. 1a–l) are arranged successively, from the superior to the inferior region of the thorax.

Image-based diagnosis can be implemented either manually by radiologists or automatically using a computerized system. Routine diagnostic tasks require the radiologists to combine information from several hundreds of sagittal, coronal and transverse CT slices. Manual methods executed by the radiologists are considered the gold standard but it requires substantial reading experience from radiologists. Since the accuracy is dependent on the experience of the radiologists, they are prone to high intra-reader and inter-reader variability. Manual methods are time consuming, labour-intensive with high risk of fatigue-induced human errors. Automatic methods are attractive to physicians as they are reproducible and can be implemented with minimal user interaction. Furthermore, automated methods facilitates physicians interpretation of images, thereby significantly reducing the time for making clinical decisions.

The benefits of automatic methods can be reaped only after overcoming several challenges. They include anatomic structures such as the colon, bowel gas, arteries, bronchioles and subcutaneous cavity that exhibit similar grayscale as the lung region. Other challenges are the uneven intensity distribution within the lung region and the presence of noise, particularly images acquired at low dose. Segmentation becomes more challenging with the presence of pathological features which increase the variability of image attributes across slices. For example, in CT images of patients with COVID-19 and interstitial lung diseases (ILD), the presence of ground glass opacity [2] and fibrosis [3], respectively, result in discontinuous lung contours which makes it difficult for automated systems to accurately extract the lung region. Some of the slices such as Fig. 1a, k and l do not contain lung region. Thus, another challenge is the reduction of false positives by the identification and exclusion of slices that do not contain the lung region.

Automatic lung region segmentation can be categorized into traditional and machine learning methods [4, 5]. The traditional methods are explicitly programmed mathematical models which include gray-scale thresholding, region growing, deformable models, wavelets and watershed techniques. The machine learning methods are models that learns the segmentation task either in an unsupervised manner from the available image data or in a supervised manner from example images. Clustering algorithms are examples of unsupervised machine learning methods. Supervised machine learning methods include deep convolutional neural networks (CNN) that are designed as a single unified model which learns both feature extraction and classification tasks [6].

None of the techniques from traditional methods can, by itself, accurately extract lung region from a CT data because the attributes of lung CT cannot be described by precise mathematical models [7]. These limitations encouraged the introduction of hybrid methods which integrates multiple traditional techniques into a three-stage system to enhance segmentation accuracy [8]. The three stages are pre-processing, processing and post-processing. In a three-stage system, there is significant increase in the number of operating parameters. Therefore, it becomes difficult to optimize segmentation accuracy across slices. A trained deep learning network, unlike the traditional methods, is a single unified model which does not require parameter tuning, thereby making it adaptable to variability of images in a clinical setting. This paper proposes a deep learning-based CT lung segmentation algorithm that follows the three-stage system adopted by the traditional methods. Three main contributions of this paper are summarized below.

1. To the best of our knowledge, the proposed method is the first application of a simple yet effective deep learning technique to implement each of the three stages of the segmentation system adopted by traditional methods. Of particular note is the introduction of a new deep learning-based method to refine the contour of lung regions at the post-postprocessing stage.
2. At the pre-processing stage, we apply a CNN to identify and exclude CT slices that do not contain lung region. This approach significantly reduces the risk of false positives in the proposed system. Also, we generate binary images from three different databases. These binary images doubles as the input training images and as the output training labels of a CNN-based U-net. The trained network converts a grayscale CT slice to a binary image. To execute the clustering task, the input training images of the U-net are grayscale images while the output training labels are k-means clustered images.
3. At the post-processing stage, binary images containing lung region and edge images of the ground truth are the training images and training labels of a U-net, respectively. The trained network corrects for pathology-induced discontinuity of lung contours. We incorporate a self verification unit within the post-processing stage by applying another CNN to identify and exclude, from the segmented image, any object that do not contain lung region.

## Related work

Traditional methods include a three-stage segmentation proposed in [9]. The first stage exploits the gray level intensity difference between the thorax region and the background. Optimal thresholding and morphology are applied to retain only the thorax region by removing the background air and CT table. Connected component analysis is executed at the second stage to extract the lung from the thorax region. In the third stage, the borders of the lung region are refined by applying morphological closing operation to insert the juxtapleural nodules and tissues that may have been excluded during the first stage. In [10], the first two stages of a three-stage segmentation of lung nodules from CT images is for the extraction of the lung region. In the first stage, a global threshold, computed from the gray level histogram of the CT slices, was used for the extraction of the preliminary lung region. A morphological closing operation was applied in the second stage to include juxtapleural nodules in segmented lung regions and to refine the contours of the lung region. In the last stage, k-means clustering is applied on the extracted lung region to detect and segment potential lung nodules. In [11], the authors propose the Selective Binary and Gaussian filtering-new Signed Pressure Force (SBGF-new SPF) function. It is based on the Chan Vese’s [12] and Geodesic [13] active contour models. Statistical information from gray level pixels are applied to avoid re-initialization and to automatically place the initial contour on the CT image. The Gaussian filter enables smooth regularization and ensures a sharper reconstructed image. The parameters of the contours are encoded in the SPF function so that the SPF can control the direction of evolution. The contribution by [8] integrates six techniques into a three-stage system. In the first stage, a guided filter smooths the image to improve contrast between different regions and remove noise. The second stage is the binarization of the image using Otsu adaptive thresholding [14]. There are three steps within the third stage. First, a region growing technique is applied to extract the thorax region from the binarized image. The second step is the application of random walk method to extract the lung from the thorax region. Finally, after morphological hole filling operation, a curvature-based correction method proposed by [15] is applied to refine the contours of the lung region. Another three-stage system was recently proposed in [16]. In the first stage, a CT slice is decomposed into four different components in the logarithmic domain. The component of interest is the shading component because it preserves lung contours and details. Image filtering is implemented by formulating the decomposition as a minimization problem. In the second stage, wavelet transformation is combined with morphological operations to extract the lung region. The last stage is the smoothing and correction of the lung contours using a corner detection technique. A three-stage system based on watershed transformation was proposed in [17]. In the first stage, gradient image of the CT slice was generated using a Sobel mask. In the second stage, the internal markers are specified, followed by morphological operation to extract the external markers. In the last stage, the markers are combined with the gradient image. Thereafter, a watershed transform is applied on the combined image to obtain the watershed lines which describe the initial lung contours. The refinement of the lung contours is implemented using a rolling ball filter to smooth the lung borders.

Deep learning techniques have been applied in a single-stage system or as a unit within a multi-stage system. In [18], the authors propose a single-stage system that utilize a deep learning technique to perform an end-to-end segmentation without any pre-processing and post-processing steps. The deep learning architecture is a U-net CNN [19], a type of fully convolutional neural network [20] with a wider network. The wide network is achieved by gradually increasing the number of kernels in the convolutional layer from 16 to 128 in the encoding path and decreasing from 128 to 8 in the decoding path. The wide network enables extraction of spatial contextual information at different levels resulting in lung region with finer details. A three-stage segmentation that utilized U-net was proposed in [21]. Cropping of the CT slices are performed in the pre-processing stage to significantly reduce the number of pixels in the background region and remove the CT table. The classical U-net was utilized in the second stage to extract the lung region mask. The mask was further applied in the third stage to segment the lung parenchyma. A three-stage segmentation system was proposed in [3, 22] to segment lung for the detection of ILD. In [22] the CT slice is preprocessed for noise removal using a Wiener filter. The processing stage is the extraction of texture features based on gray level co-concurrence matrix and deep features from U-net. In the postprocessing stage, the texture and deep learning features are fused to derive the contours of the lung region. In the contribution by [23], the preprocessing step is image normalization. The processing step applies U-net architecture to extract the lung region. The postprocessing stage utilize Gaussian kernel to remove noise, followed by morphological operation to remove unwanted structures that do not belong to the lung region.

## Materials and method

### Description of data

This study utilizes retrospectively acquired CT scans, in DICOM format, from four databases. They are the Lung Image Database Consortium (LIDC-IDRI) image collection [24], 3DIRCAD (3D Image Reconstruction for Comparison of Algorithm Database) [25] and the Interstitial Lung Diseases (ILD) database [26]. The fourth database (PHTM) consist of realistic phantoms. The LIDC-IDRI has 1,018 helical thoracic CT scans from seven academic centres. The 3DIRCAD contains two databases. The first, 3D-IRCAD-01 and second, 3D-IRCAD-02 contains anonymized CT scans of 20 and 2 patients, respectively, with expert-annotated binary masks of thoracic organs. Six binary masks of the lung are provided in 3D-IRCAD-01. The data of both patients in 3D-IRCAD-02 include binary masks of the lung. The ILD database was provided by the University Hospital of Geneva. It contains high-resolution computed tomography (HRCT) images of 128 patients affected with ILD. The data has annotated lung tissue patterns including lung mask as well as a comprehensive set of 99 clinical parameters related to ILDs. The PHTM contains 10000 images generated from the Kyoto Kagaku Lungman phantom (http://kyotokagaku.com/products/detail03/pdf/ph-1_manual.pdf) at the department of radiology and nuclear medicine, Oslo University Hospital. The mother phantom has a main body height 45 cm and circumference 94 cm with provisions for extensions to simulate bigger size patients. Furthermore, it is composed of a chest wall, mediastinum and an abdominal block. The phantom was initially utilized in the study of the effect of iterative reconstruction levels on image quality [27].

### Deep learning architecture

The algorithm was built from two classical CNNs (CNN-1 and CNN-2) and two U-net CNNs (UNET-1 and UNET-2) deep learning models that are widely used for image classification and segmentation, respectively. The CNN consists of three successive stages of convolutions with 8, 16 and 32 convolution layers of size $3 \times 3$, respectively. The convolution layer at each stage is stacked with rectifier linear unit, batch normalization layer and max pooling layer. The rectifier linear unit operates like a thresholding algorithm which outputs the input directly if it is positive, otherwise, it outputs zero. The batch normalization layer normalizes the activations and gradients propagating through the network while the max pooling layer reduces the spatial size. Features learned from the stack is connected to a fully connected layer which contains softmax activation function for classification.

The U-net is structured into down-sampling and up-sampling sections. The down-sampling structure is a stack of two convolution layers, max pooling layer and rectifier linear unit which down-samples its input by a factor of 4 with no padding. The up-sampling structure is a stack of two transposed convolution layers and rectifier linear unit which increases the size of the input from the down-sampling structure by a factor of 4. Each convolution layer and the transposed convolution layer have 64 filters of size $3 \times 3$.

The CNN-1 will be at the preprocessing stage to predict whether or not the input image contains a lung region. The UNET-1 and UNET-2 will perform segmentation and lung contour refinement, respectively. The CNN-2 is at the postprocessing stage to filter out none-lung regions that were erroneously segmented by UNET-1.

### Generation of training data

The accuracy and robustness of a learning technique is dependent on the availability of example images that captures a wide range of image attributes that is commonly found in a clinical setting. Thus, the training phase of deep learning models requires reasonably large volume of labeled CT data which is a labour-intensive task for radiologists. Our strategy to address this problem will follow the recent work of the authors in [28]. Their design philosophy is based on the assumption that the lung region presents common visual and geometric similarities across subjects, diseases and CT scanners. They propose a weakly supervised approach to generate a large amount of CT images from unlabeled data, which is subsequently used to train and validate the CNN model. The trained model was evaluated on another separate set of expert-annotated labeled CT data.

**Fig. 2:**
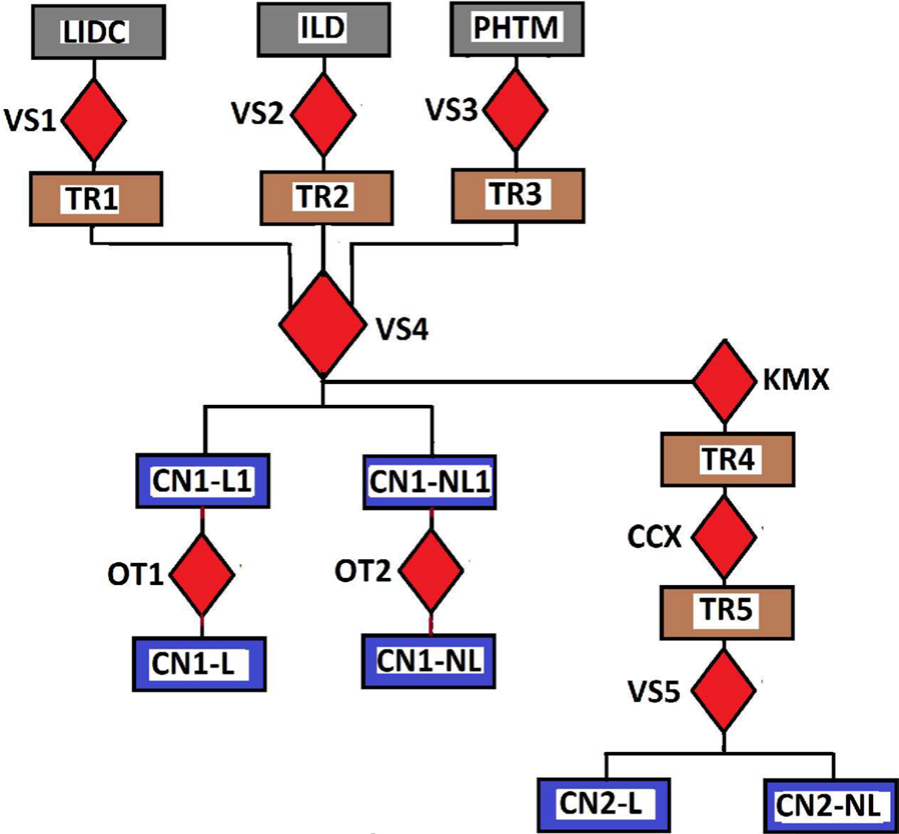
Flow chart for the generation of training data for CNN-1 and CNN-2 models. Three databases **LIDR**, **PHTM** and **ILD** were visually inspected (**VS1**, **VS2**, **VS3**) and three sets of grayscale images (**TR1**, **TR2**, **TR3**) was selected. These images are combined to into a single set and visually inspected (**VS4**) again to categorize them into images containing lung region **CN1-L1** and images without lung region **CN1-NL1**. Otsu thresholding **OTX** is applied to images in each category which converts them to binary images and tagged training data, **CN1-L** and **CN1-NL** for CNN-1. The generation of training data for CNN-2 begins with applying k-means **KMX** clustering to the sets of images (**TR1**, **TR2** and **TR3**) from the three databases. Application of connected component analysis **CCX** on the output **TR4** of the clustering algorithm produces set of images **TR5** which are visually inspected **VS5** and categorized into components containing lung region **CN2-L** and components without lung region **CN2-NL**

Figure 2 shows the flow chart for generating the training data for two CNN models (CNN-1 and CNN-2). It begins with visual inspection (**VS1**, **VS2**, **VS3**) of three databases (**LIDC**, **PHTM** and **ILD**). During the inspection, three sets of grayscale images (**TR1**, **TR2**, **TR3**) are selected. These images are combined into a single set and visually inspected (**VS4**) again to categorize them into images containing lung region **CN1-L1** and images without lung region **CN1-NL1**. Application of Otsu thresholding (**OT1,** **OT2**) on each category of images converts them to a training data consisting of lung region **CN1-L** and none lung region **CN1-NL**. The generation of training data for CNN-2 begins with clustering **KMX** the three sets (**TR1**, **TR2** and **TR3**) with k-means-based clustering tree [29]. A clustering tree is clustering at multiple clustering resolutions [30]. Clustering tree does not require the user to specify a predefined number of cluster, so it overcomes a major limitation of k-means clustering. This is followed by analysis of connected component **CCX** of the output **TR4** of the clustering algorithm. This produce new set of images **TR5**. The new set of images are visually inspected **VS5** to generate training data which is categorized into lung region **CN2-L** and none lung region **CN2-NL**.

In this study there are five different configurations of training data (see Table 1) for the U-net model. In the first approach, expert-annotated binary masks of lung regions and its duplicate copy are the training images and the training labels, respectively. The training images and the training labels in the second configuration are binary images generated using Otsu-based threshold method. The foreground of CT lung images doubles as the training images and the training labels in the third configuration. The fourth and fifth configurations of training data are generated by applying k-means clustering to segment grayscale images, from the three databases, into two and three classes. The grayscale images and the k-means clustered images are the training images and training labels, respectively, of the U-net model.

**Table 1:** The five different configurations of the training data

Training data	
Training images	Training labels
Ground truth images	Ground truth images
Otsu-based binary images	Otsu-based binary images
Foreground images	Foreground images
Grayscale images	Two-class k-means clustered images
Grayscale images	Three-class k-means clustered images

**Fig. 3:**
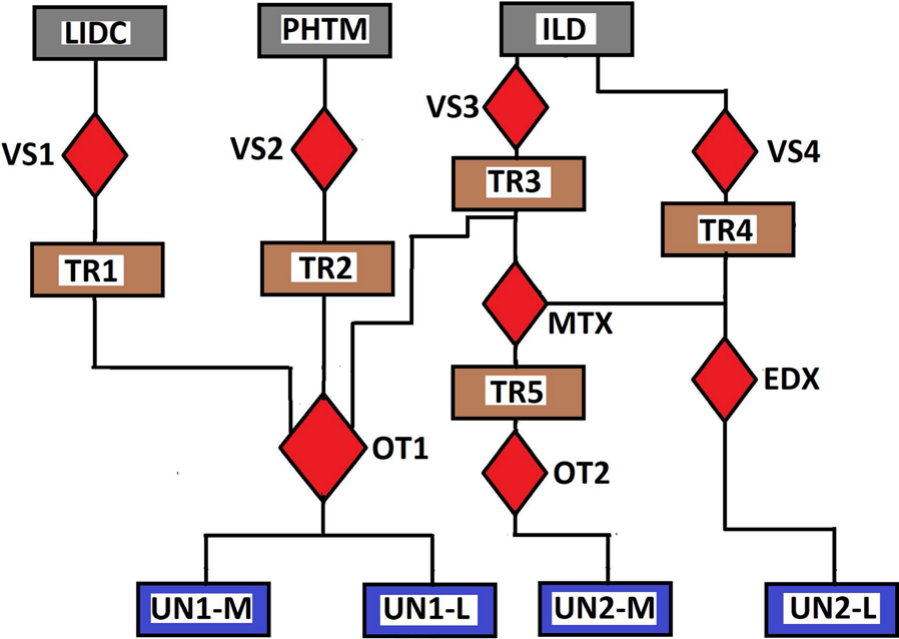
Flow chart for generating the second configuration of training data for UNET-1 and the training data for UNET-2 models. Three databases **LIDR**, **PHTM** and **ILD** were visually inspected (**VS1**, **VS2**, **VS3**) to select three sets of grayscale images (**TR1**, **TR2**, **TR3**). Otsu thresholding **OT1** converts the three sets of images into binary images. The binary images are combined into a single set and further classified into two categories; the training images **UN1-M** and training labels **UN1-L** for the training of UNET-1. The ILD database is again visually inspected **VS4** to select a set of expert-annotated masks **TR4** that corresponds to grayscale images in **TR3**. The corresponding images in **TR3** is multiplied **MTX** with **TR4** to obtain new set of images **TR5**. The **TR5** is converted to a binary image using Otsu thresholding **OT2** and are tagged the training images **UN2-M** for UNET-2. Canny edge detector **EDX** extract edge images from **TR4** and these edge images are tagged the training labels **UN2-L** for UNET-2

Figure 3 displays the flow chart for generating the second configurations of training data for UNET-1 and the training data for UNET-2. Three databases **LIDC**, **PHTM** and **ILD** were visually inspected (**VS1**, **VS2**, **VS3**) to select three sets of grayscale images (**TR1**, **TR2**, **TR3**). Otsu thresholding **OT1** converts the three sets of images into binary images. The binary images are combined into a single set tagged **UN1-M**, the training images for UNET-1. We make a duplicate copy of **UN1-M** and tagged it as **UN1-L**, the training labels for UNET-1. The **ILD** database is again visually inspected **VS4** to select a set of expert-annotated masks **TR4** that corresponds to grayscale images in **TR3**. The corresponding images in **TR3** is multiplied **MTX** with **TR4** to obtain new set of images **TR5**. The **TR5** is converted to a binary image using Otsu thresholding **OT2** and are tagged the training images **UN2-M** for UNET-2. Canny edge detector **EDX** extracts edge images from **TR4** and these edge images are tagged with the training labels **UN2-L** for UNET-2.

### Training

The proposed method was implemented using the MATLAB computing environment on a Microsoft Windows 10 Education edition personal computer. The system has an installed physical memory of 16 GB with Intel(R) Core(TM) processor rated as i7-8650U CPU @ 1.90GHz, 2112 Mhz. For all the CNNs and U-nets, the initial learning rate and maximum epochs was set at 0.001 and 100, respectively. The validation frequency and mini batch size for the CNNs and U-nets are 30 and 32, respectively.

**Fig. 4:**
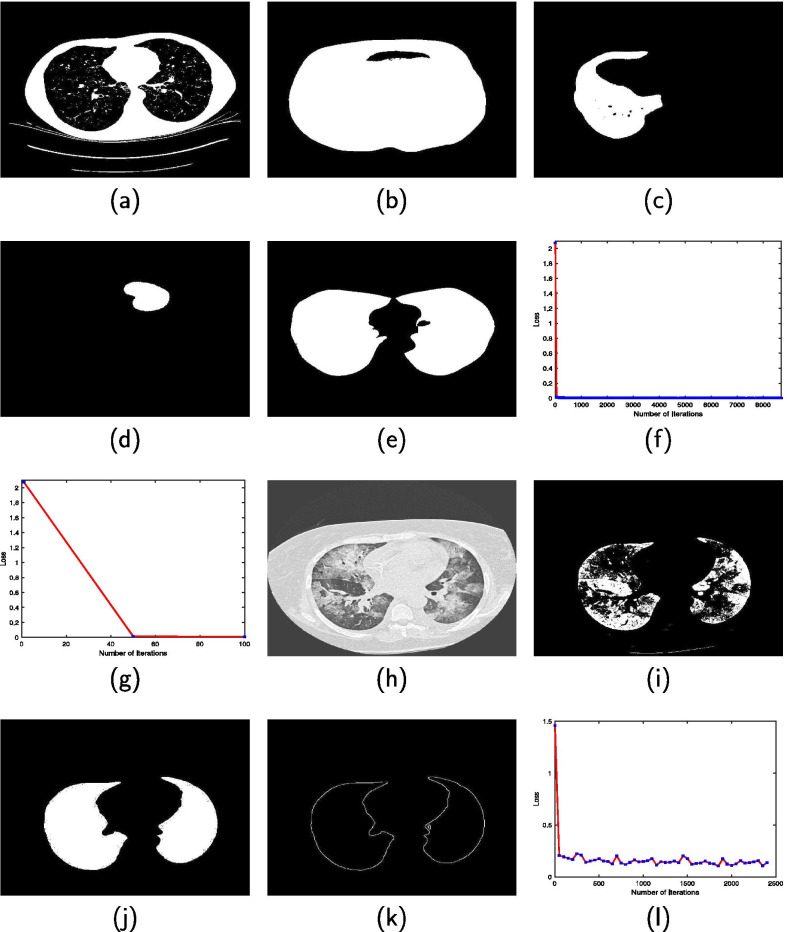
Binary image tagged as **a** containing and **b** not containing lung region in the CNN-1 training data. Connected component tagged as **c** containing and **d** not containing lung region in the CNN-2 training data. **e** Binary mask of a grayscale CT slice that doubles as training image and training label in UNET-1 training data. **f** Plot of loss versus number of iterations during the training of UNET-1. **g** Zoomed-in version of the plot in the preceding subfigure. **h** CT slice from the ILD database. **i** Binary image of the slice in the preceding subfigure. **j** Lung region of the CT slice from the ILD database. **k** Contour image of the lung region shown in the preceding subfigure. **l** Plot of loss versus number of iterations during the training of UNET-2

The training data for CNN-1 model contains 5000 binary images each for the class of lung region and none lung region. Figure 4a and b are examples of the binary image containing lung and none regions, respectively. The CNN-2 model utilize 10,000 training data. Examples of images that contain connected components belonging to the lung and none lung regions are in Fig. 4c and d, respectively. The length of the training phase are 3 and 5 h for CNN-1 and CNN-2, respectively, and accuracy of 99.6 and 97.0%, respectively.

Four thousand binary images, each for the training images and the training labels, were utilized to train the first U-net model. Figure 4e is example of a binary mask that is tagged as both a training image and training label. Figure 4f is a plot of loss versus number of iterations during the training phase of the first U-net model. A zoomed-in version (Fig. 4g) of the plot in Fig. 4f shows that it takes approximately 50 iterations to attain optimal accuracy. Figure 4h is a grayscale image from the ILD database that was converted to a binary image (Fig. 4i) and used as one of the 780 training images for training the second U-net model. The image in Fig. 4j is the binary mask of Fig. 4h that was converted to edge image (Fig. 4k) which is tagged as a training label in the training data of the second U-net model. In Fig. 4l, the plot of loss versus number of iterations show that the training phase of the second U-net model attained optimal accuracy in less than 50 iterations.

**Fig. 5:**
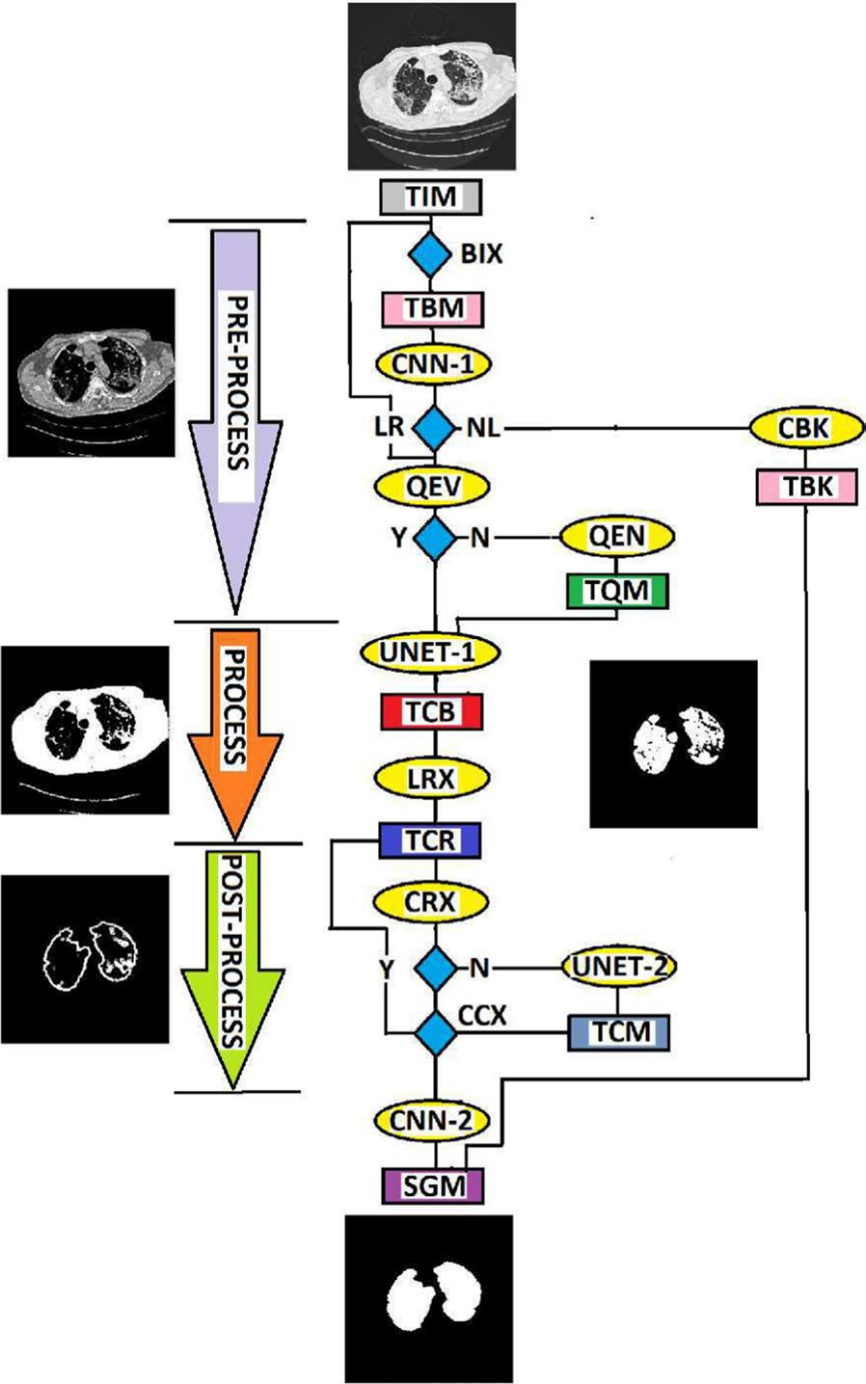
The three successive steps of the proposed lung region segmentation. **Preprocessing**: The CNN-1 identifies only the CT slices that contains lung region followed by quality evaluation. **Processing**: The identified CT slice is fed to a UNET-1 which converts the slice to a binary image. **Postprocessing**: Each connected components in the binary image are fed to CNN-2 and UNET-2 to extract only connected components associated with the lung region and for contour refinement, respectively, before the segmented image is displayed

**Fig. 6:**
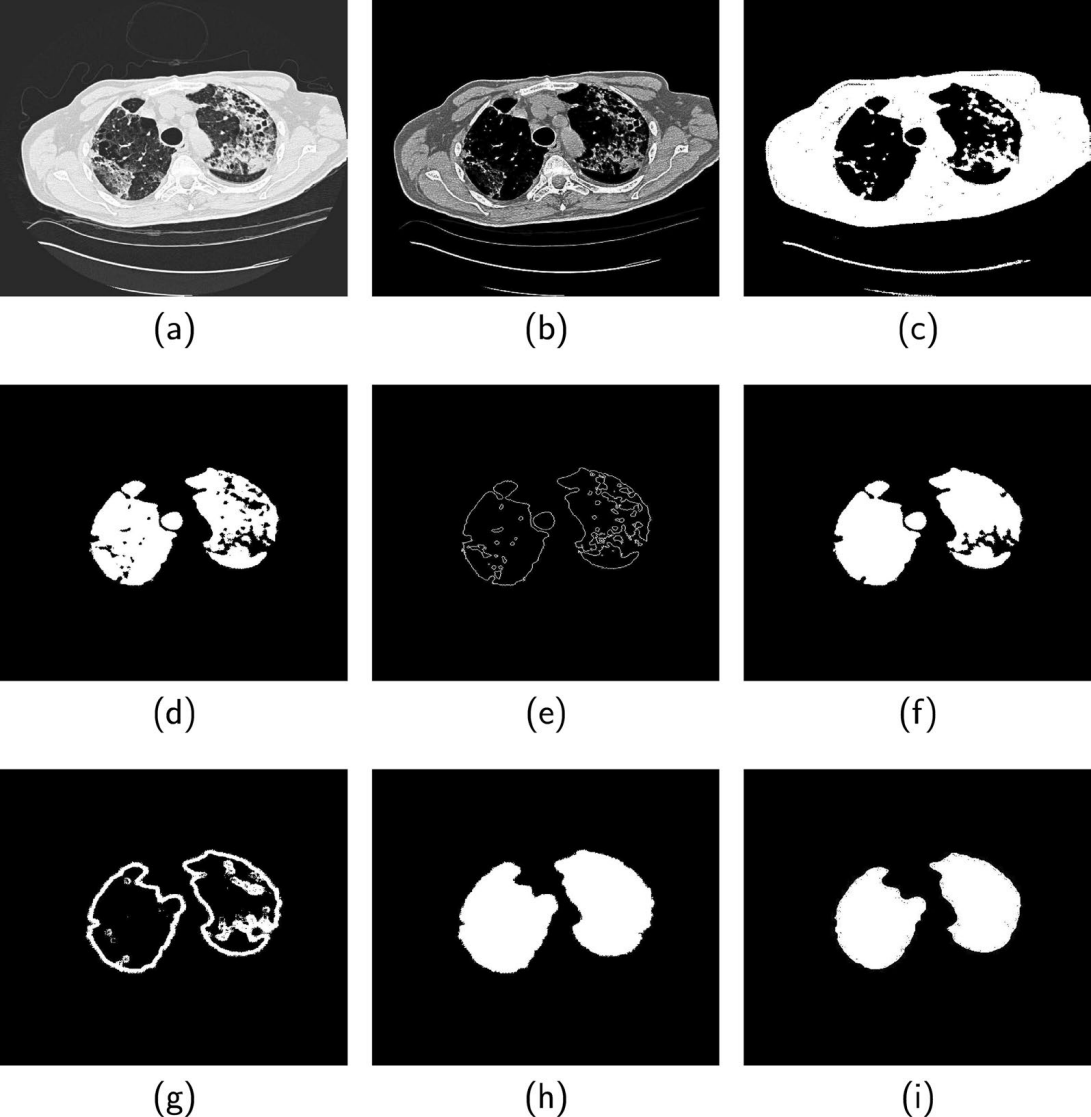
Implementation of the proposed method using image from the ILD database. **a** Original image. **b** Contrast enhancement of the original image. **c** Binary image of the original image. **d** Candidate lung region. **e** Perimeter feature of the binary image. **f** Area feature of the original image. **g** Refined contour. **h** Segmented image. **i** Ground truth

### Segmentation

The flow chart in Fig. 5 and the images in Fig. 6 describe the successive steps to implement the proposed three-stage segmentation system. Detailed implementation are provided below.

#### Pre-processing

The Otsu thresholding algorithm is applied on the test image **TIM** shown in Fig. 6a. This step converts **BIX** to a binary image **BIM** before it is fed to a classifier **CNN-1**. If the classifier predicts **NL** that the image do not contain a lung region, a function **CBK** generates an image **TBK** having only zero pixels as the segmented slice **SGM**. For prediction **LR** which indicate the presence of lung region, the quality of the test image is evaluated **QEV** using our previous contribution [31]. We set quality score of 0.9 as threshold $\tau _{1}$ to determine whether to enhance **Y** or not to enhance **N** the contrast quality of the test image before it is moved further for processing. The quality score of the test image is 0.81, hence the system apply contrast enhancement algorithm **QEN** proposed by [32] on the test image. We set the enhancement algorithm tuning parameter at $\beta =4$. The contrast-enhanced image **TQM** in Fig. 6b is moved to the processing stage.

#### Processing

At the processing stage, a trained U-net **UNET-1** converts the grayscale test image to a binary image **TCB** shown in Fig. 6c. The binary image is further fed to a function **LCR** which extract candidate lung region **TCR** shown in Fig. 6d. The candidate lung region are the dark pixels in Fig. 6a that lies within the foreground region of the grayscale image.

#### Post-processing

The image **TCR** that contains the candidate lung region is moved to a function **CRX** which computes a variable $\tau _{2}$ that determines whether to refine **N** or not to refine **Y** the borders of the extracted lung region. The **CRX** function incorporates a Canny edge detector to compute edge image (Fig. 6e) of the extracted lung region and a morphological operator (Fig. 6f) to fill holes in the extracted lung region. We define $\tau _{2}=A_{2}/ A_{1}$ where $A_{1}$, $A_{2}$ are the areas of the edge image and the filled image in Fig. 6e and Fig. 6f, respectively. The reasoning behind the definition of $\tau _{2}$ is that correctly extracted lung regions have completely filled regions with smooth and closed contours. Therefore, $\tau _{2}$ will generally increase with the quality of the smoothness and contours that define the lung region. We set a threshold $\tau _{2}=12$, but $A_{2}/ A_{1}=10$ for the image under consideration. Therefore, the candidate lung region **TCR** is fed to a trained network **UNET-2** which refines the contours of the lung borders. The output of the trained network **TCM** shown in Fig. 6g is filed with holes and each region in the filled image is identified using connected component analysis **CCX** and fed to a classifier **CNN-2**. The classifier performs extra self-verification on the image by filtering out none lung regions and passing on only regions considered as lung to form the segmented image **SGM** in Fig. 6h.

#### Evaluation metric

The accuracy of the segmentation was evaluated using the Dice similarity coefficient *D*. Specifically, all images segmented using UNET-1 trained on the five different configurations of the training data were evaluated based on the dice similarity score with respect to their corresponding ground truth. The dice score is a popular statistical tool used for the validation of deep learning-based image segmentation algorithms. It measures the similarity between the segmented region *X* and the ground truth *Y* based on how they overlap [33].

1 $$\begin{aligned} D(X,Y)=\frac{2|X \cap Y|}{|X| + |Y|} \end{aligned}$$

## Experiments and results

There were two experiments to evaluate the performance of the proposed method. The first experiment utilizes 1230 slices from the 3DIRCAD database. The second utilize 1100 slices from the ILD database. Tables 2 and 3 display the performance evaluation results for each experiment based on the Dice score. The first and second columns of Tables 1 and 2 are five different configurations of the training data. The remaining columns are the three different configurations of our segmentation system.

**Table 2:** 3DIRCAD database: performance of the proposed method using five configurations of training data at the processing stage and three configurations of the segmentation system

Training data		Dice score		
Training images	Training labels	Three-stage system	Without CNNs	Without contrast enhancement
Ground truth images	Ground truth images	0.81	0.76	0.79
Otsu-based binary images	Otsu-based binary images	0.85	0.81	0.80
Foreground images	Foreground images	0.83	0.77	0.80
Grayscale images	Two-class k-means clustered images	0.90	0.81	0.80
Grayscale images	Three-class k-means clustered images	0.92	0.83	0.82

**Table 3:** ILD database: performance of the proposed method using five configurations of training data and three configurations of the segmentation system

Training data		Dice score		
Training images	Training labels	Three-stage system	Without CNNs	Without Contrast Enhancement
Ground truth images	Ground truth images	0.87	0.85	0.81
Otsu-based binary images	Otsu-based binary images	0.92	0.91	0.83
Foreground images	Foreground images	0.88	0.84	0.79
Grayscale images	Two-class k-means clustered images	0.93	0.87	0.85
Grayscale images	Three-class k-means clustered images	0.95	0.86	0.87

**Fig. 7:**
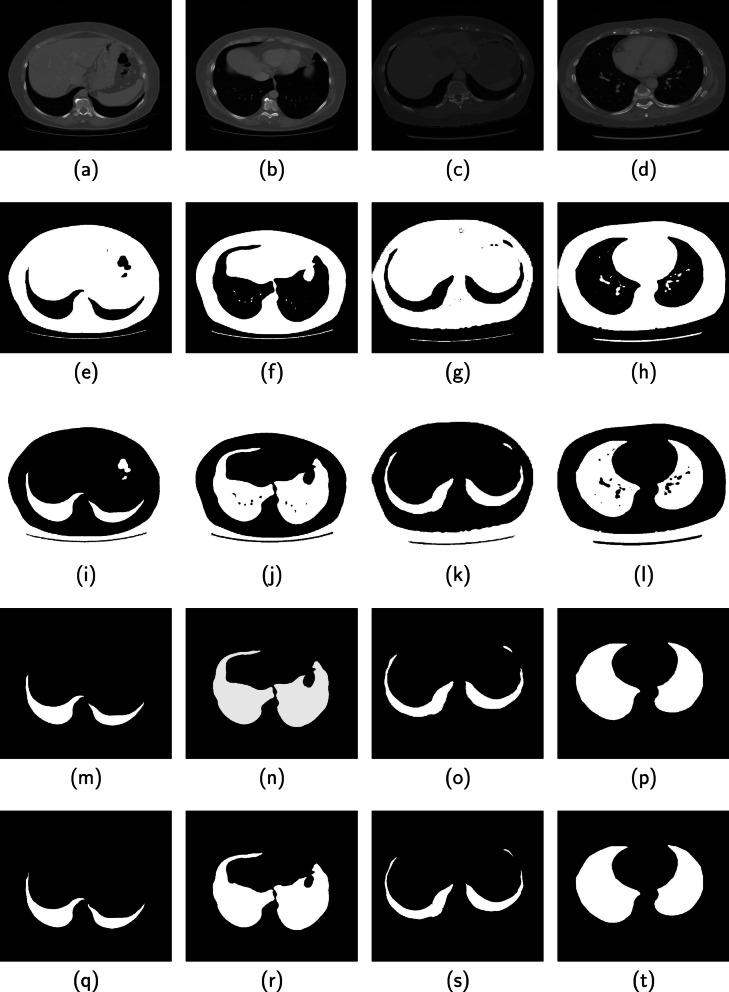
Performance of the proposed method on four CT images from the 3DIRCAD database are displayed in columns 1, 2, 3 and 4. (Row 1) Original image, (Row 2) Binarized image, (Row 3) Candidate lung region, (Row 4) Segmented lung region (Row 5) Ground truth

**Fig. 8:**
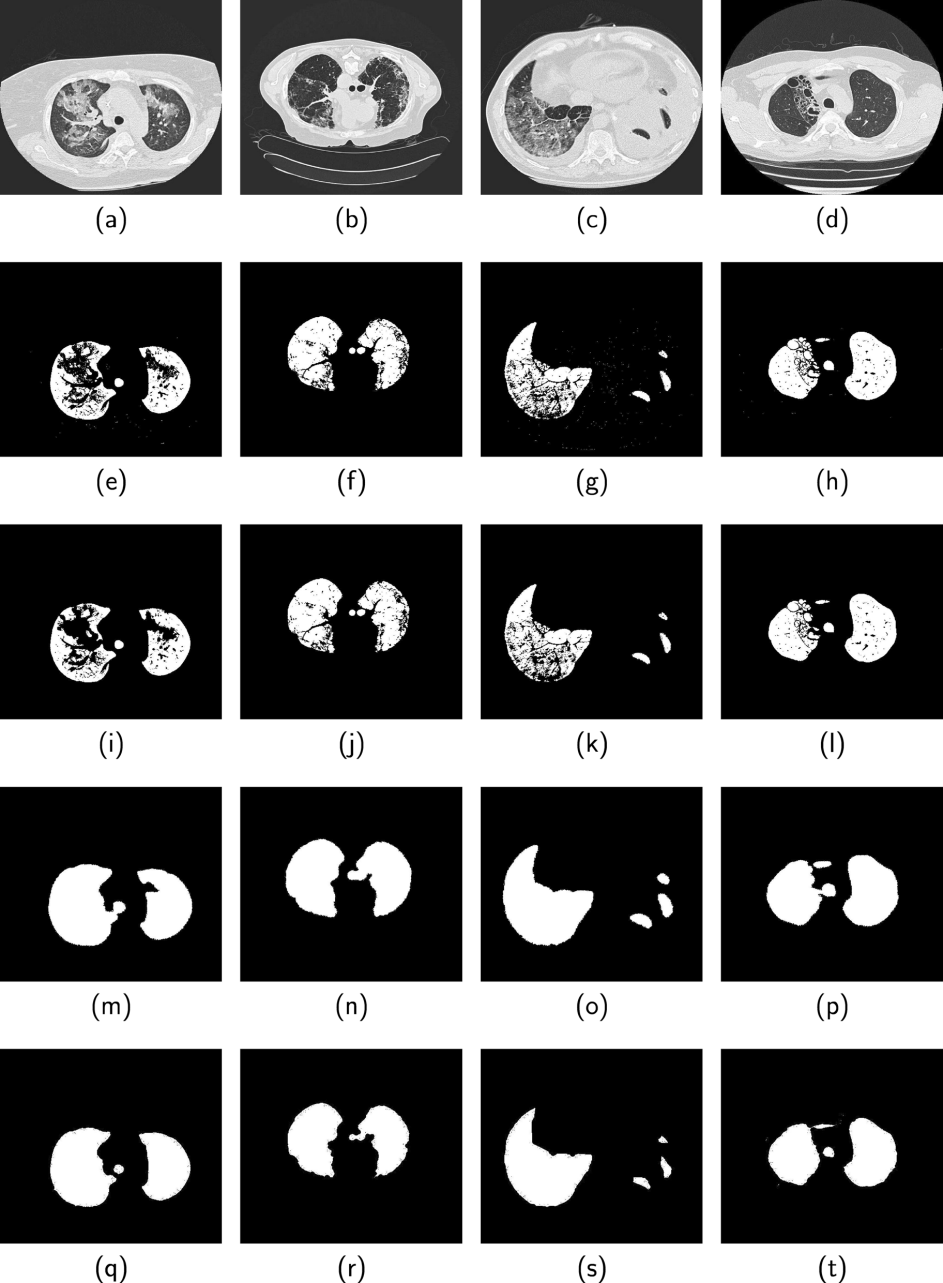
Performance of the proposed method on four CT images from the ILD database are displayed in columns 1, 2, 3 and 4. (Row 1) Original image, (Row 2) Binarized image, (Row 3) Candidate lung region, (Row 4) Segmented lung region (Row 5) Groundtruth

**Fig. 9:**
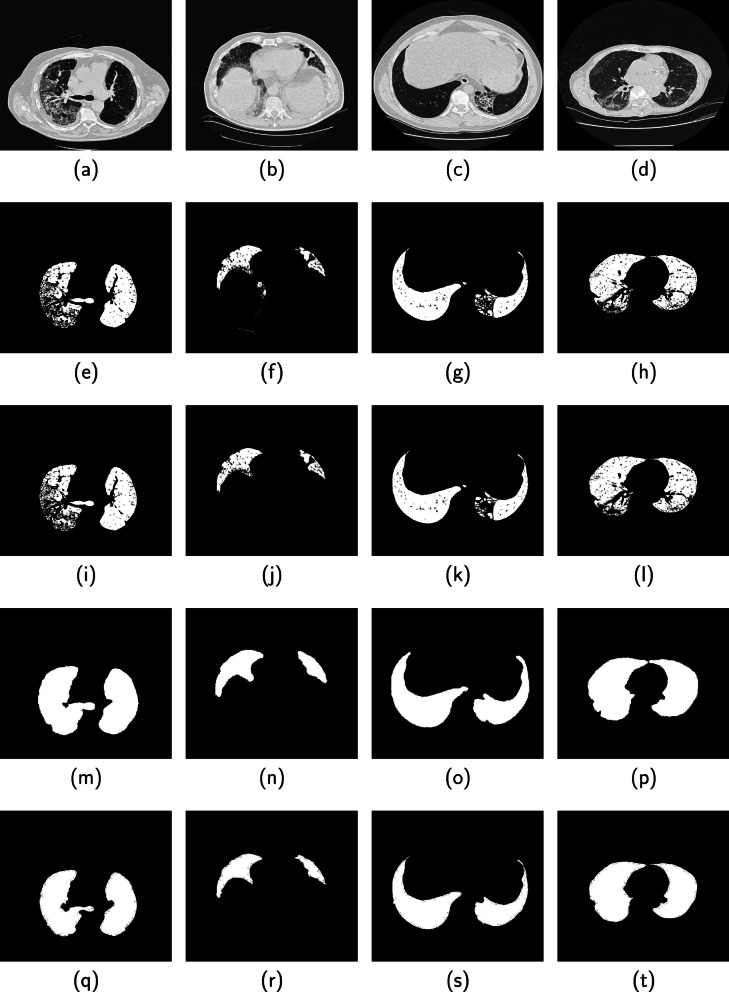
Another performance of the proposed method on four CT images from the ILD database are displayed in columns 1, 2, 3 and 4. (Row 1) Original image, (Row 2) Binarized image, (Row 3) Candidate lung region, (Row 4) Segmented lung region (Row 5) Groundtruth

Four images displayed in each column of Fig. 7 are slices from the 3DIRCAD database. The second, third, fourth and fifth rows in each column are the binarized image, candidate lung region, segmented lung region and ground truth. Two sets of four images from the ILD database are displayed separately in Figs. 8 and 9. The binarized image, candidate lung region, segmented lung and the ground truth are displayed in the second, third, fourth and fifth row in each column of the respective figures.

**Fig. 10:**
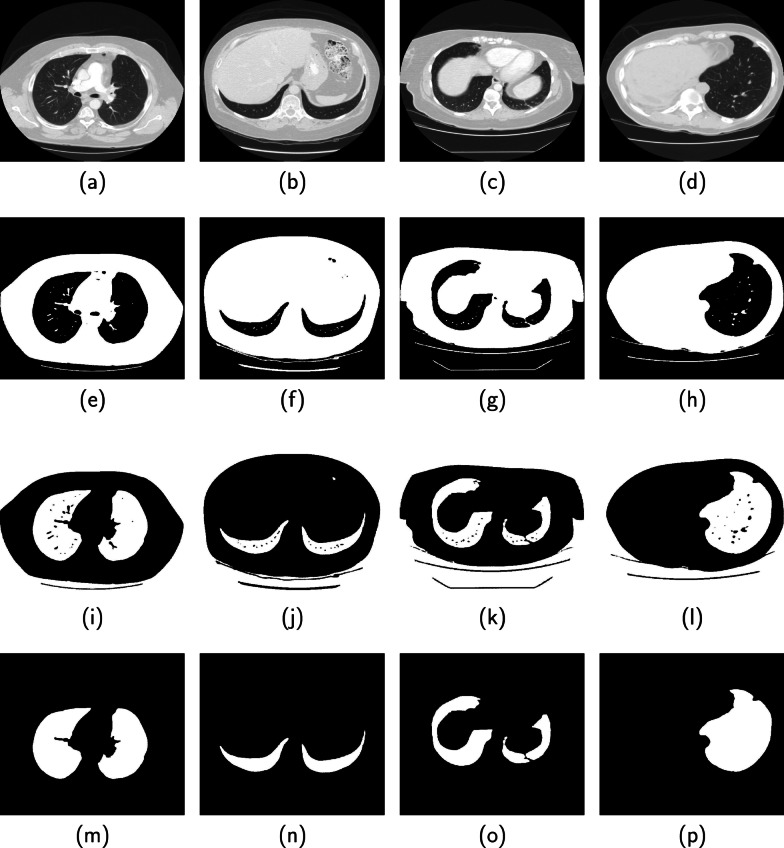
Images describing the performance of the proposed method on four CT images from the LIDC database are displayed in columns 1, 2, 3 and 4. (Row 1) Original image, (Row 2) Binarized image, (Row 3) Candidate lung region, (Row 4) Segmented lung region

**Fig. 11:**
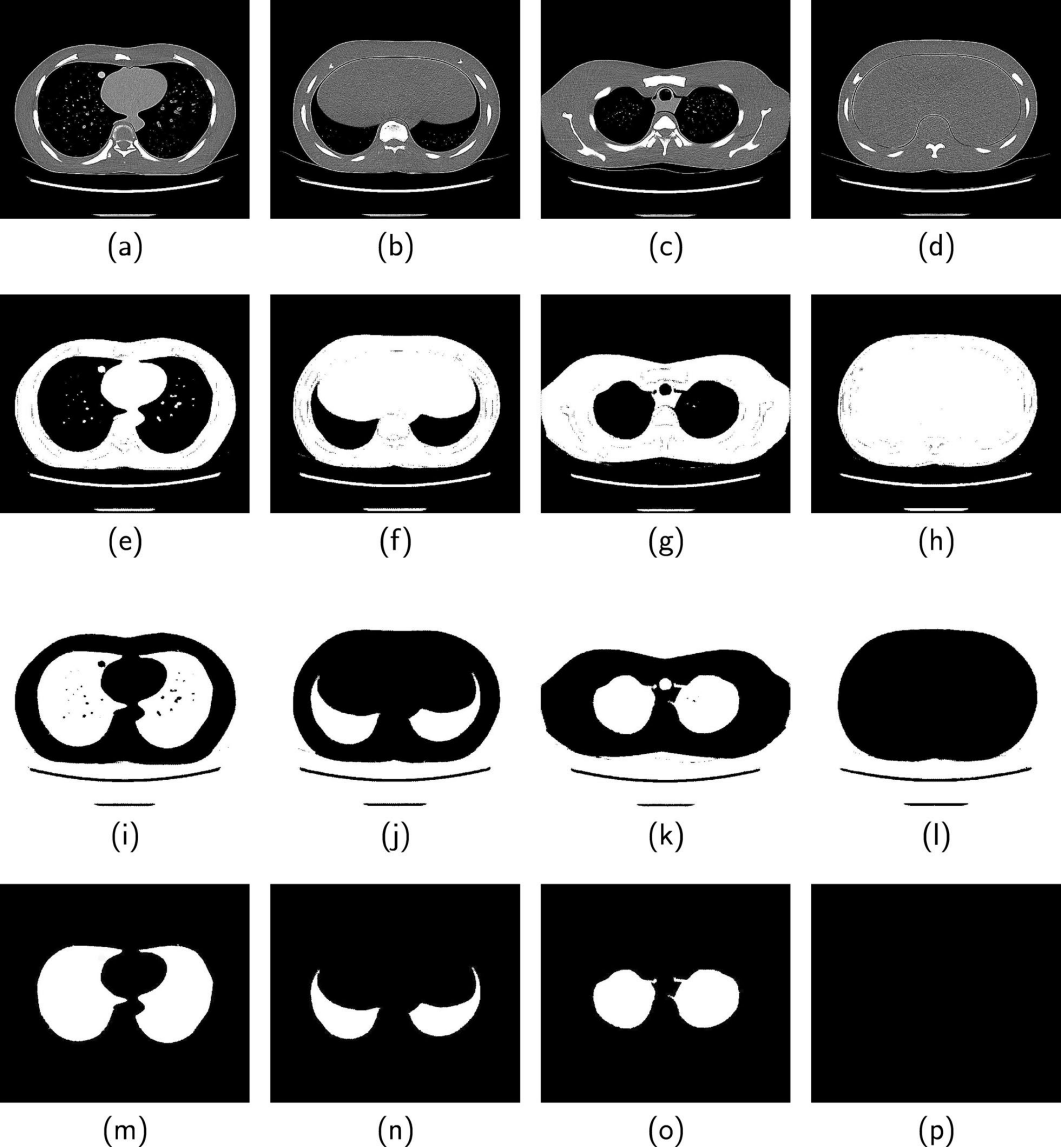
Images describing the performance of the proposed method on four CT images from the PHTM database are displayed in columns 1, 2, 3 and 4. (Row 1) Original image, (Row 2) Binarized image, (Row 3) Candidate lung region, (Row 4) Segmented lung region

The proposed method was also evaluated on 500 and 700 images from the LIDC and the phantom databases. Ground truth are not available for these two databases, however, we display four test images from each database in each column of Figs. 10 and 11. The second, third, fourth and fifth rows in each column of these figures are the corresponding binarized image, candidate lung region, segmented lung and the ground truth images.

## Discussion

In the past five years, artificial intelligence (AI) has demonstrated potential as a subspecialty in the filed of radiology and medicine by providing useful tools to facilitate physician’s interpretation of images for the diagnosis of diseases [34]. Deep learning-based AI generally demonstrate better performance than traditional image processing methods and has pushed the limits for machine learning, artificial intelligence and computer vision in many applications [35, 36]. Trained deep learning models, unlike the traditional methods, have relatively few operating parameters. Traditional methods formulated as a three-stage system have relatively large operating parameters, thereby making it difficult to optimize segmentation accuracy across images with different attributes. For this reason, there is growing interest among researchers to apply deep neural network in the implementation of the different steps within traditional methods. The authors in [37] and [38] apply deep CNN techniques at the preprocessing stage for the removal of noise in low dose CT images. An instance segmentation technique inspired by the intuitive and classical watershed transform was proposed in [39]. The authors use deep CNN to develop a model that generates a modified watershed energy landscape from which high quality objects can be extracted. The authors in [40] propose a multilevel wavelet CNN. Their design philosophy is to embed wavelet transform into a CNN architecture to simultaneously reduce the resolution of feature maps and increase receptive field. Following on this trend, we implemented the three stages of the traditional methods using deep neural networks. Below, we outline several design features and the performance of the proposed method which makes it a useful tool in a clinical setting.

### Importance of quality evaluation and enhancement

Image analysis is a critical step in the evaluation of chest CT radiological characteristics and lesion distribution patterns in patients of respiratory diseases such as COVID-19 pneumonia [41]. Most current contributions ignore quality evaluation of the training images whereas the reliability of diagnosis is strongly dependent on image quality. The proposed method incorporates a no-reference image quality evaluation and the quality of a CT slice can be enhanced when its quality score is below a predefined threshold. This approach reduces the manual task of subjective evaluation by radiologists and significantly reduces the time it takes for experts with differing opinions on image quality to arrive at a consensus. Columns 3 and 5 in Tables 1 and 2 are the dice scores recorded by the proposed method with and without quality evaluation and enhancement, respectively. Results from the experiments shows that image quality evaluation and enhancement can improve segmentation accuracy.

### Simple and efficient algorithm

We utilize simplified versions of CNNs and U-nets to implement each stage of the segmentation system. Images at the processing stage are binary images. The proposed method can be considered simple, fast, computationally efficient with reproducible segmentation results. Therefore, it is potentially a valuable and priceless friend rather than a foe of radiologists as it can assist radiologists to overcome challenges encountered in the diagnosis of diseases so that they have more time attending to patients [42].

### Exploring different configurations of training data

Our proposed method explore five different approaches to configure a training data. The five different configurations of training images and training labels are displayed in the first and second columns of Tables 1 and 2. Limited training time was recorded for training data configurations shown in the first, second and third rows. The plot of loss versus number of iterations for the training data containing only ground truth images (first row of Table 2) is displayed in Fig. 4f. The plot shows that optimal accuracy is attained in less than 50 iterations, approximately 15 min. Two factors contribute to limit the length of the training phase. First, all the training data are in the binary domain which is naturally computationally efficient. Second, the training labels are duplicate copy of the training images. Thus, our proposed method can be considered as a step towards the application of deep learning to real-time operations.

### Deep learning-based contour refinement

The presence of ground-glass opacities in COVID-19 patients [2] and fibrosis in ILD patients [3] causes discontinuity in the border of lungs in CT images. This feature makes it difficult for automated systems to accurately extract the lung region. To the best of our knowledge, current approaches are based on traditional image processing methods. For example, the contribution by [3] refines the candidate lung region by correcting the contour of the left lungs using the Smallest Univalue Segment Assimilating Nucleus (SUSAN) algorithm [43] and repairing the regions of the right lungs. Another contribution [44] that propose to segment multiple organs of the thoracic cavity using U-net-generative adversarial network (U-net-GAN) refines the lung using morphological operations. Traditional methods are naturally prone to errors in the presence of varying image attributes. The proposed deep learning-based method potentially overcomes the limitations of traditional methods as it learns the attributes of examples images with pathology during the training phase.

### Cheap and quality data

The implementation of deep learning-based AI requires reasonable large volume of example images from which the model can learn patterns and features [45]. Local, regional and international regulations on patient privacy makes it difficult to obtain enough data for the implementation of deep learning-based AI. Furthermore, the generation of large volume of labeled data for training is a daunting and expensive task as it requires the recruitment of several radiologists. This paper addresses the problem of data availability by utilizing k-means clustering to automatically generate cheap and large volume of labeled CT data across different databases to augment limited training data. Review of the generated data by radiologists will require minimal manual labour.

### Reduction of false positives

The efficacy of automated image analysis for the diagnosis and treatment of respiratory disease such as COVID-19 can be compromised by presence of intensity inhomogeneity, artifacts, and closeness in the gray level of different soft tissues in the thoracic region [46]. All the slices acquired during CT examination do not contain lung regions. Examples are CT slices located towards the inferior and superior regions of the thorax. Some diagnostic task require manual identification and exclusion of slices containing none lung regions before the useful slices are fed to a automated system. This paper eliminates manual task by the use of a CNN classifier at the pre-processing stage to identify and exclude slices that do not contain lung region. Another CNN classifier located at the post-processing stage is an extra verification step to reduce false positives and enhance segmentation accuracy. Comparison of Dice scores in the third and fourth columns of Tables 1 and 2 show that the use of CNNs can reduce false positives and increase segmentation accuracy.

### Comparative performance evaluation

We utilize three publicly available databases for this study. Two of the databases, the 3DIRCAD and the ILD databases have ground truths but the images in both databases were not categorized into training and test sets. Several researchers who utilized the databases for segmentation arbitrarily selected their training and evaluation data from the databases. For this reason, it was impossible to have a common dataset for a fair quantitative comparison of our proposed method with the algorithms proposed by other authors.

### Limitations and future work

The proposed paper does not address all the challenges which limits the deployment of AI in a clinical setting. For example, the deep learning algorithm does not include explainable AI that can provide insight on how and why the model makes a decision at different stages of the segmentation. Also, we did not involve the services of a radiologist to evaluate the accuracy of the automatically generated images that were used to augment the training data. Future research direction will incorporate explainable AI and explore the use of pre-trained models to enhance segmentation accuracy.

## Conclusions

Traditional image processing methods are considered efficient because they incorporate explicit mathematical models that can easily be optimized for a single or limited number of images. However, its performance can be limited by the variability of attributes across large volumes of images in a clinical setting. The integration of several traditional techniques with the aim of improving performance increases the algorithm operating parameters, thereby making it difficult to optimize the algorithm operating performance and segmentation accuracy. This paper exploit the benefits of deep learning techniques by proposing a three-stage segmentation where each stage of the segmentation is implemented using deep learning technique. The trained network at the different stages of the segmentation significantly reduces the algorithm operating parameters by learning the variations in image attributes from example images across three different databases. The proposed method was evaluated on images from two publicly available databases and the results are promising for application in a clinical setting.

## Data Availability

The data used for this study are from three publicly available databases and a realistic phantom. They are the Lung Image Database Consortium (LIDC-IDRI) image collection [24], 3DIRCAD (3D Image Reconstruction for Comparison of Algorithm Database) [25] and the Interstitial Lung Diseases (ILD) database [26] The websites to download the data are LIDC-IDRI https://wiki.cancerimagingarchive.net/display/Public/LIDC-IDRI 3DIRCAD https://www.ircad.fr/research/3dircadb/ ILD http://medgift.hevs.ch/wordpress/databases/ild-database/ The realistic phantom data can be obtain on request from the corresponding author.

## Abbreviations

AI: Artificial intelligence
COVID-19: Corona virus disease 2019
CT: Computed tomography
HRCT: High resolution computed tomography
CNN: Convolutional neural network
U-net: U-net convolutional neural network
SBGF-new SPF: Selective binary and Gaussian filtering-new signed pressure force
DICOM: Digital imaging and communications in medicine
LIDC-IDRI: Lung image database consortium
3DIRCAD: 3D Image reconstruction for comparison of algorithm database
ILD: Interstitial lung disease
SUSAN: Smallest univalue segment assimilating nucleus
U-net-GAN: U-net generative adversarial network
